# In Vitro and In Vivo Response of Zinc-Containing Mesoporous Bioactive Glasses in a Sheep Animal Model

**DOI:** 10.3390/ijms232213918

**Published:** 2022-11-11

**Authors:** Javier Jiménez-Holguín, Daniel Arcos, Daniel Lozano, Melchor Saiz-Pardo, David de Pablo, Luis Ortega, Silvia Enciso, Blanca Fernández-Tomé, Idoia Díaz-Güemes, Francisco Miguel Sánchez-Margallo, Laura Casarrubios, María Teresa Portolés, María Vallet-Regí

**Affiliations:** 1 Departamento de Química en Ciencias Farmacéuticas, Facultad de Farmacia, Universidad Complutense de Madrid, Instituto de Investigación Sanitaria Hospital 12 de Octubre i+12, Plaza Ramón y Cajal s/n, 28040 Madrid, Spain; 2 CIBER de Bioingeniería, Biomateriales y Nanomedicina, CIBER-BBN, ISCIII, 28040 Madrid, Spain; 3 Servicio de Anatomía Patológica, Hospital Clínico San Carlos, Facultad de Medicina, Universidad Complutense de Madrid, Instituto de Investigación Sanitaria Hospital Clínico San Carlos (IdISSC), 28040 Madrid, Spain; 4 Centro de Cirugía de Mínima Invasión Jesús Usón, NANBIOSIS, 10071 Cáceres, Spain; 5 Departamento de Bioquímica y Biología Molecular, Facultad de Ciencias Químicas, Universidad Complutense de Madrid, Instituto de Investigación Sanitaria del Hospital Clínico San Carlos (IdISSC), 28040 Madrid, Spain

**Keywords:** mesoporous bioactive glass, zinc, bone regeneration, bioactivity, cell cultures, in vivo study

## Abstract

Zinc-enriched mesoporous bioactive glasses (MBGs) are bioceramics with potential antibacterial and osteogenic properties. However, few assays have been performed to study these properties in animal models. In this study, MBGs enriched with up to 5% ZnO were synthesized, physicochemically characterized, and evaluated for their osteogenic activity both in vitro and in vivo. The ZnO MBGs showed excellent textural properties despite ZnO incorporation. However, the release of Zn^2+^ ions inhibited the mineralization process when immersed in simulated body fluid. In vitro assays showed significantly higher values of viability and expression of early markers of cell differentiation and angiogenesis in a ZnO-content-dependent manner. The next step was to study the osteogenic potential in a sheep bone defect model. Despite their excellent textural properties and cellular response in vitro, the ZnO MBGs were not able to integrate into the bone tissue, which can be explained in terms of inhibition of the mineralization process caused by Zn^2+^ ions. This work highlights the need to develop nanostructured materials for bone regeneration that can mineralize to interact with bone tissue and induce the processes of implant acceptance, cell colonization by osteogenic cells, and regeneration of lost bone tissue.

## 1. Introduction

Since their discovery in 2004 by Yan et al. [1], mesoporous bioactive glasses (MBGs) have become one of the most interesting bioceramics in the last two decades due to their potential application as grafts for regenerative treatments of bone tissue [2,3,4,5]. MBGs are bioactive glasses that, depending on the organic template used, can have different mesoporous structures and exhibit a much higher specific surface area and porosity than conventional sol-gel glasses [6]. These excellent textural properties facilitate rapid ion exchange with the surrounding medium, leading to the rapid and extensive formation of a nanocrystalline apatite phase very similar to the mineral component of bone [7]. Consequently, MBGs have a higher in vitro bioactivity than other bioactive glasses when in contact with solutions that mimic the inorganic fraction of human plasma, such as simulated body fluid (SBF) [8]. Moreover, the ordered mesoporous structure facilitates their application as local drug delivery systems [9] and even as stimuli-responsive systems for on-demand drug release [10]. From the point of view of in vivo behavior, these materials present excellent properties to regenerate bone defects, both as powders [11] and as scaffolds [12], as well as in combination with polymers or other compounds that control their high degradation rate in the physiological environment [13].

The incorporation of therapeutic ions in the form of oxides has been one of the most developed aspects in the field of MBGs [14]. Thus, different ions have been incorporated into the SiO_2_–CaO–P_2_O_5_ system that could endow these materials with angiogenic, antimicrobial, anti-inflammatory or even osteogenic properties, providing new benefits to these bioceramics [15,16,17]. Among the different ions with potential therapeutic properties, Zn^2+^ ion has been one of the most studied for incorporation into MBGs. In this regard, zinc-containing MBGs have been prepared in the form of macroporous scaffolds [18], nanoparticles [19,20,21,22], coatings [23,24], and composite fibrous membranes [25]. Zn^2+^ ions have shown antibacterial properties in vitro when they are part of MBGs in the SiO_2_–CaO–P_2_O_5_–ZnO system [26,27,28]. In addition, Zn^2+^ stimulates the differentiation of osteoprogenitor cells towards the mature osteoblast phenotype by regulating the expression of alkaline phosphatase (ALP), osteopontin, and osteocalcin [29], so a potential osteogenic activity has been attributed to this ion, which makes it very interesting for bone regeneration purposes [30,31,32,33].

Despite these interesting properties evidenced under in vitro conditions, different research teams have shown that the presence of Zn^2+^ ions in the MBGs composition produces a decrease and even inhibits the crystallization of hydroxyapatite on the surface of these kind of materials [26,32,34,35,36,37]. According to the classical mechanism proposed by Hench [38] for SiO_2_-based glasses, the formation of an apatite-like phase is necessary for bone bonding to occur, as well as subsequent tissue regeneration as the glass dissolves. Thus, Zn^2+^ ions could favor osteogenesis by stimulating osteoprogenitor differentiation but, at the same time, inhibit this process by not allowing HA formation on the glass surface, which contributes to the adhesion of osteoprogenitor cells to the material.

Despite the abundant scientific literature published on zinc-containing MBGs, there is a surprising paucity of in vivo studies that shed light on this aspect [39]. In this article, we have prepared a series of compositions in the SiO_2_–CaO–P_2_O_5_–ZnO system that have been subjected to an in-depth physicochemical and structural study, as well as an evaluation of the in vitro behavior, both in SBF and with preosteoblast cultures. Finally, the in vivo response to these materials has been evaluated in a sheep animal model, establishing a direct comparison between MBGs with and without ZnO in their composition.

## 2. Results

**Figure 1:**
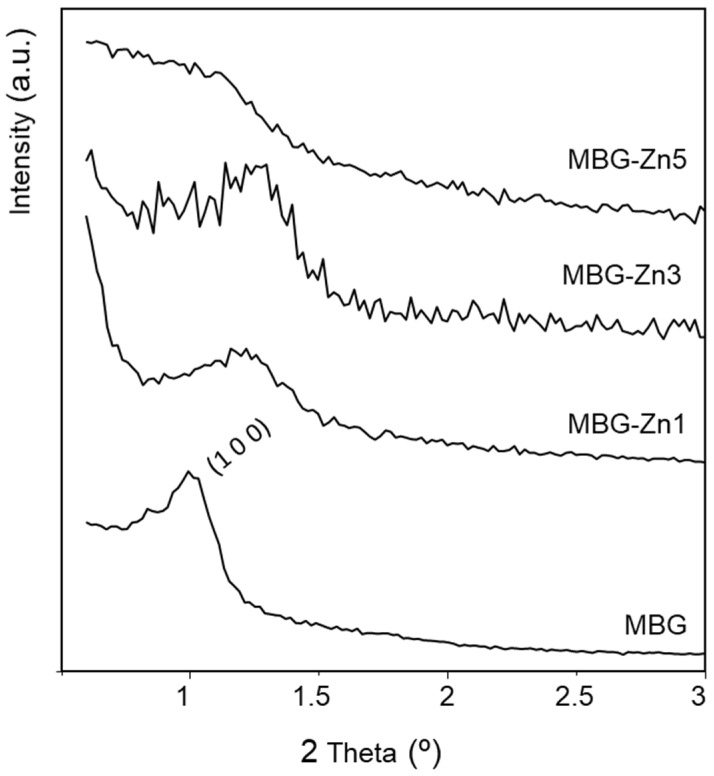
Low angle XRD patterns for MBG and Zn-containing MBGs.

The study of the mesoporous order of the MBGs with and without ZnO was carried out by XRD (Figure 1). The patterns indicate a loss of structural ordering insofar as the content of ZnO increases. In this sense, the MBG sample exhibits a well-resolved diffraction maxima at 2θ° = 1.0 that can be assigned to the (1 0 0) reflection of a *p6m* 2D hexagonal group. This maximum becomes less resolved for MBG-Zn1 and MBG-Zn3 and disappears for MBG-Zn5. Besides, the lattice parameter, a_0_, decreases with the ZnO content as can be deduced from the shift of the unique diffraction maxima towards higher 2θ° angles.

**Figure 2:**
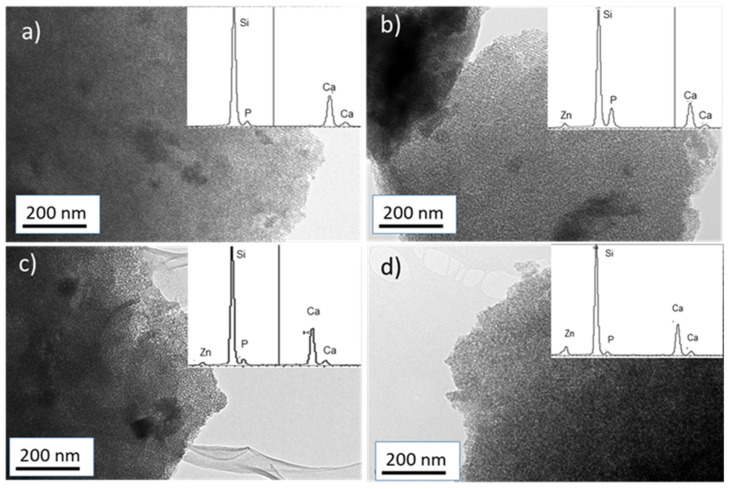
TEM images and EDX spectra obtained for (**a**) MBG, (**b**) MBG-Zn1, (**c**) MBG-Zn3, and (**d**) MBG-Zn5.

TEM images (Figure 2) show the presence of mesopores in the four synthesized samples. In the case of MBG and MBG-Zn1, an ordered channel structure compatible with a 2D hexagonal structure is clearly observed (Figure 2a,b). However, the samples with higher ZnO content present a less ordered mesoporous (worm-like) structure, in agreement with the loss of ordering observed by XRD. The EDX spectra collected during TEM observation indicate that the chemical composition of the MBGs is very similar to the calculated nominal compositions (Table 1). 

**Figure 3:**
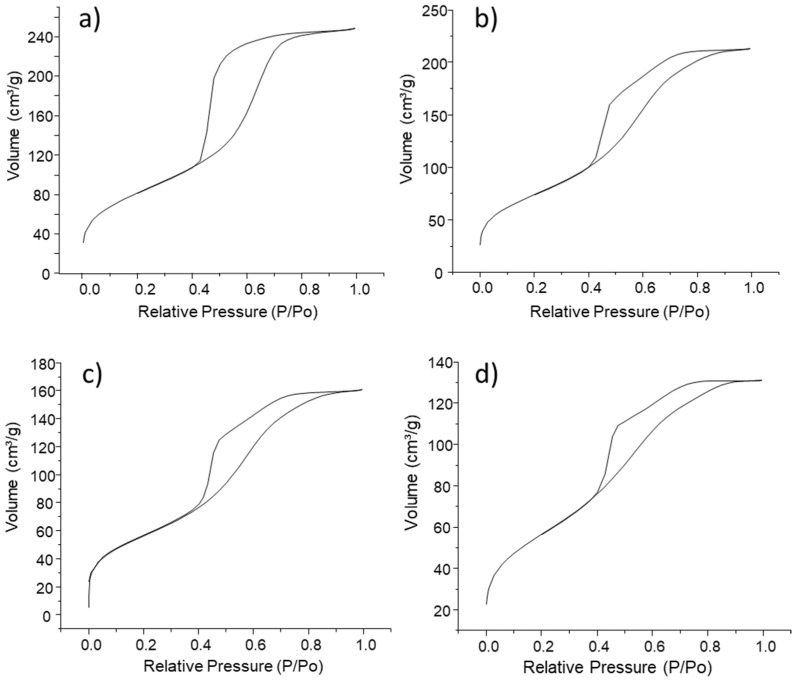
Adsorption/desorption of N_2_ isotherms obtained for (**a**) MBG, (**b**) MBG-Zn1, (**c**) MBG-Zn3, and (**d**) MBG-Zn5.

N_2_ adsorption analysis provides very interesting information about the evolution of the textural properties of the MBGs as a function of ZnO content. Figure 3 shows the adsorption/desorption isotherms for the four compositions studied. The MBG (Figure 3a) shows a type IV isotherm characteristic of mesoporous materials with a hysteresis loop H1, indicating the cylindrical morphology of the mesopores [40]. ZnO-containing MBGs also exhibit type IV adsorption isotherms. However, the asymmetry of the hysteresis loop due to the modification of the slope of the desorption isotherm indicates that the presence of ZnO leads to the appearance of secondary porosity, which would be detrimental to mesoporous ordering. In fact, the surface area, pore volume, and pore size decrease as the ZnO content increases, as can be seen in Table 2. 

The analysis of the pore size by N_2_ adsorption analysis together with the pore-to-pore distance, a_0_, calculated by XRD allows us to calculate the wall thickness of the different MBGs (see Table 2). In this sense, we can observe that there is a general contraction of the structure of the MBGs insofar as ZnO content increases. 

**Figure 4:**
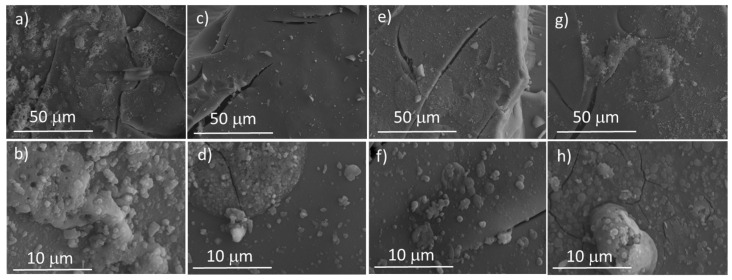
SEM micrographs obtained after 0 and 24 h in SBF for (**a**,**b**) MBG, (**c**,**d**) MBG-Zn1, (**e**,**f**) MBG-Zn3 and (**g**,**h**) MBG-Zn5.

The in vitro bioactivity of Zn-substituted MBGs was evaluated by reacting the materials in SBF and analyzing the formation of a carbonate–apatite phase on the surface of the glasses. Figure 4 shows the SEM micrographs of the MBGs before and after being immersed for 24 h in SBF. All the MBGs analyzed showed the formation of a new phase consisting of spherical particles of about 0.5–2 µm in size, which could correspond to the early nucleation of a newly formed calcium phosphate.

**Figure 5:**
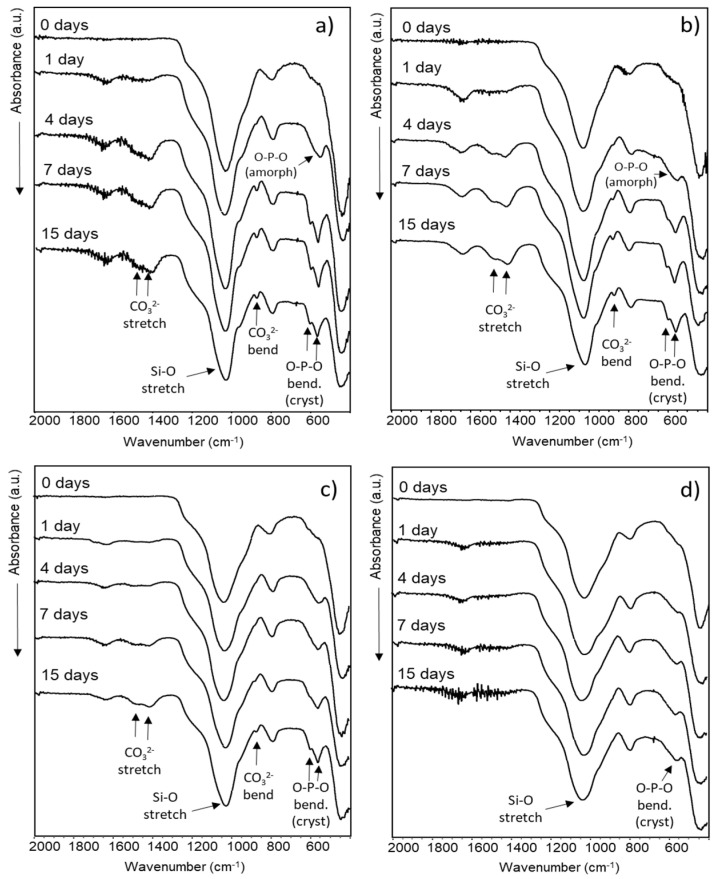
FTIR spectra of (**a**) MBG, (**b**) MBG-Zn1, (**c**) MBG-Zn3, and (**d**) MBG-Zn5 collected as a function of soaking time in SBF.

Figure 5 shows the FTIR spectra of the MBGs obtained before and after being treated with SBF for 1, 4, 7, and 15 days. Before reacting with SBF, all MBGs show absorption bands at 1030 cm^−1^ and 450 cm^−1^ corresponding to the Si-O stretching and O-Si-O bending modes, respectively. The in vitro mineralization process was verified by the initial nucleation of an amorphous calcium phosphate and the subsequent crystallization of an apatite-like phase. Nucleation of amorphous calcium phosphate was verified by the appearance of a singlet around 590 cm^−1^, corresponding to the O-P-O bending mode of PO_4_^3−^ anions in the amorphous state, while the crystallization of the apatite phase was followed by the evolution of this singlet towards a doublet signal characteristic of phosphate ions in a crystalline environment. In addition, the occurrence of absorption bands around 1465 cm^−1^ and 870 cm^−1^ corresponding to the stretching and bending vibrational modes, respectively, of the CO_3_^2−^ groups were analyzed. MBG, MBG-Zn1, and MBG-Zn3 developed a carbonate–apatite crystalline phase after 4, 7, and 15 days in SBF, respectively. MBG-Zn5 only showed the formation of a weak singlet corresponding to the formation of amorphous calcium phosphate without the presence of carbonate groups. 

**Figure 6:**
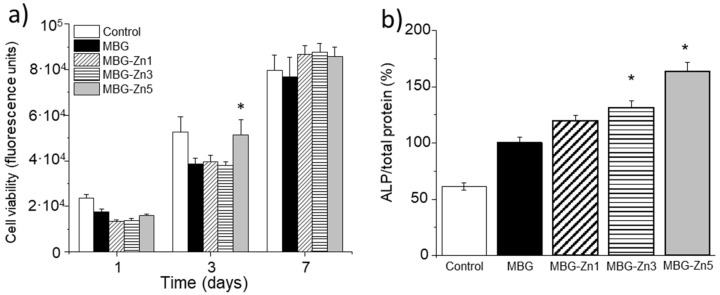
MC3T3-E1 preosteoblast viability at 1, 3, and 7 days (**a**) and ALP activity after 7 days of culture (**b**) in the presence of MBG and ZnO-containing MBGs. * *p* < 0.05.

The studies with cell cultures were carried out with MC3T3-E1 preosteoblasts, with the aim of establishing the response of these cells as a function of the ZnO content contained in the different materials. Figure 6a shows the cell viability of MC3T3-E1 cells in contact with the different materials after 1, 3, and 7 days of culture. The results show that MBG-Zn5 induced a significant increase in cell viability at day 3 compared to MBG, while the MBG-Zn1 and MBG-Zn3 materials showed no significant difference with respect to MBG. This effect stabilized at 7 days, when the cells were already confluent. Figure 6b shows the ALP activity after 7 days of culture. ALP activity increased significantly in a ZnO-dependent manner with the ZnO content in the MBG, obtaining maximum differentiation values for MBG-Zn5. On the other hand, MC3T3-E1 preosteoblast cells showed a favorable phenotype typical of this cell lineage, which is characterized by an extended cytoskeleton, a spindle-shaped morphology, and a spherical nucleus, without showing significant differences between MBGs and ZnO-containing MBGS (see Figure S4 in supporting information).

**Figure 7:**
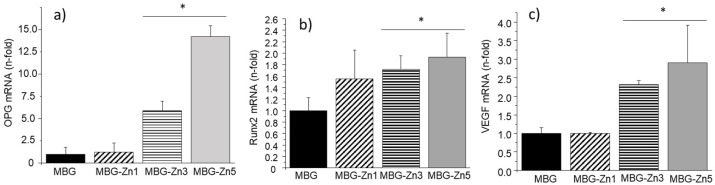
MC3T3-E1 preosteoblast gene expression (measured by qRT-PCR) of OPG (**a**), Runx2 (**b**), and VEGF (**c**) markers in the presence of MBG and ZnO-containing MBGs. * Comparison between MBG (blank) and MBG-Zn3/MBG-Zn5. Statistical significance: * *p* < 0.05.

Figure 7 shows the gene expression of different osteogenic and angiogenic markers at 10 days of culture. Similar to what was observed with ALP activity, a direct relationship of gene expression of osteoblastic differentiation markers (OPG and RUNX2) as well as angiogenesis (VEGF) was observed as a function of the ZnO content of the MBGs. In this sense, the samples of MBG-Zn3 and MBG-Zn5 induced a statistically significant increase in expression with respect to MBG, obtaining for MBG-Zn5 the highest expression values for the three markers analyzed. 

**Figure 8:**
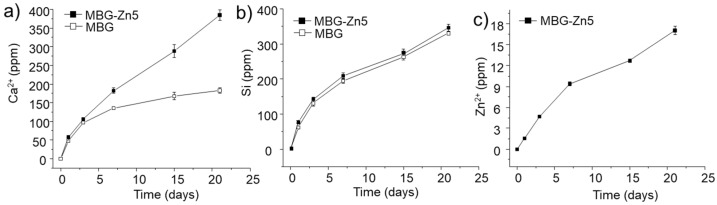
Concentration of Ca (**a**), Si (**b**), and Zn (**c**) as a function of soaking time in α-MEM obtained by ICP spectroscopy for MBG and MBG-Zn5.

In light of the results obtained for sample MBG-Zn5 with respect to viability, differentiation, and expression of osteogenic markers in osteoprogenitor cells, we decided to carry out in vivo studies comparing the Zn-free MBG with MBG-Zn5. In this way, we could obtain valuable information about the effects of introducing Zn^2+^ cations in the composition of the SiO_2_–CaO–P_2_O_5_ ternary MBGs. Prior to material implantation, we studied the ion release in culture media as a function of time. Figure 8 shows the concentration of calcium, silicon, and zinc measured by ICP spectroscopy. The lower concentration of Ca^2+^ measured for MBG after 7 days of testing is attributed to the in vitro bioactivity of this sample, which involves the precipitation of Ca^2+^ cations as carbonate hydroxyapatite on the surface, as bioactive glasses capable of forming apatite in SBF also do so in MEM (see Figure S5 in supporting information). On the contrary, MBG-Zn5 releases Ca^2+^ in an almost linear way, showing that no calcium precipitation occurs in this sample, in agreement with the in vitro bioactivity test previously described. Regarding silicon release, Figure 8b shows very similar profiles for MBG and MBG-Zn5 without observing significant differences at any time point. Finally, the zinc release measured for MBG-Zn5 indicates an accumulated concentration of zinc increase of 1.5 ppm per day during the first three days, reaching a final concentration 17 ppm after 21 days of testing. 

**Figure 9:**
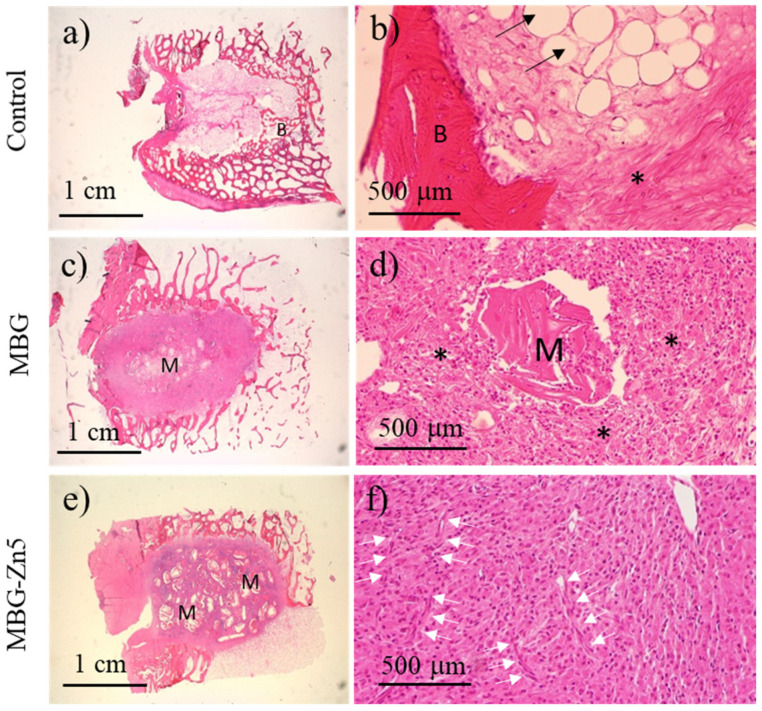
Histological images 6 weeks after implantation of control (**a** and **b**), MBG (**c** and **d**), and MBG-Zn5 (**e** and **f**) (left column: magnification ×3; right column: magnification ×20). (B) Bone tissue. (*) Inflammatory component. (M) Implanted material. (Black arrows) adipocytes. (White arrows) Blood vessels.

Figure 9 shows the histological images obtained 6 weeks after implantation in sheep bone. Control defect (Figure 9a,b) appears mainly filled by bone adipocytes (black arrows) and a mild presence of inflammatory component (asterisk), with a very low presence of new bone formation (B) and constrained to the deepest area of the defect. The histological image of MBG samples (Figure 9c,d) shows a marked presence of inflammatory component in the defect. Partially degraded particles of MBG material (M) can be observed (Figure 9c), which are mainly surrounded by polymorphonuclear cells without apposition of new bone. The histological image for MBG-Zn5 does not show reparative mineralized bone, and only material granules surrounded by inflammatory components could be observed (Figure 9e). Similar to MBG, the newly formed tissue mainly consists of polymorphonucleated and histiocyte cells, although a higher presence of blood vessels (white arrows) could be observed in all the defects implanted with MBG-Zn5 material. 

**Figure 10:**
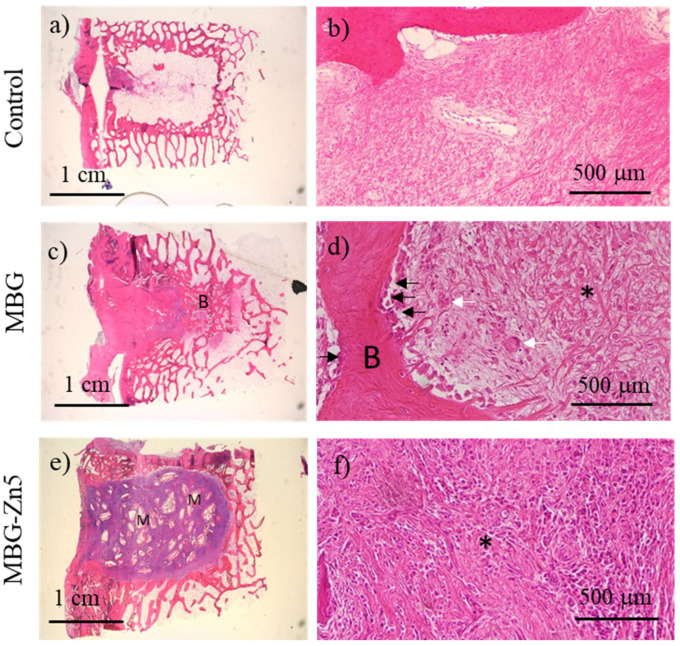
Histological images 12 weeks after implantation of control (**a** and **b**), MBG (**c** and **d**), and MBG-Zn5 (**e** and **f**) (left column: magnification ×3; right column: magnification ×20). (B) Bone. (Black arrows) osteoblasts. (White arrows) osteoclasts. (M) Implanted material. (*) Inflammatory component.

The histological images 12 weeks after implantation evidence that control defects are not repaired by new bone formation (Figure 10a,b), which indicates that the cavitary defects carried out in this model are critical enough that bone regeneration is not possible after 12 weeks. MBG samples evidence the formation of new bone tissue (B) that partially substitute the inflammatory component (Figure 10c,d) without the presence of MBG material that seems to have been resorbed. This new bone formation takes place in the deepest part of the defect. The new trabeculae show osteoblast lining (black arrows) and the presence of osteoclasts (white arrows), indicating an active process of bone remodeling in this part of the defect. On the contrary, the location closest to the drill incision remains colonized by the inflammatory component (asterisk). Finally, defects implanted with MBG-Zn5 do not show new bone formation (Figure 10e,f). Moreover, the presence of osteoblasts or osteoclasts could not be observed, whereas the inflammatory component colonizes the defect area, showing a very similar scenario to that observed after six weeks. It must be highlighted that granules of MBG-Zn5 can be clearly observed after 12 weeks (M), evidencing that this material could not be resorbed and remains in the defect site surrounded by fibrous tissue. 

**Figure 11:**
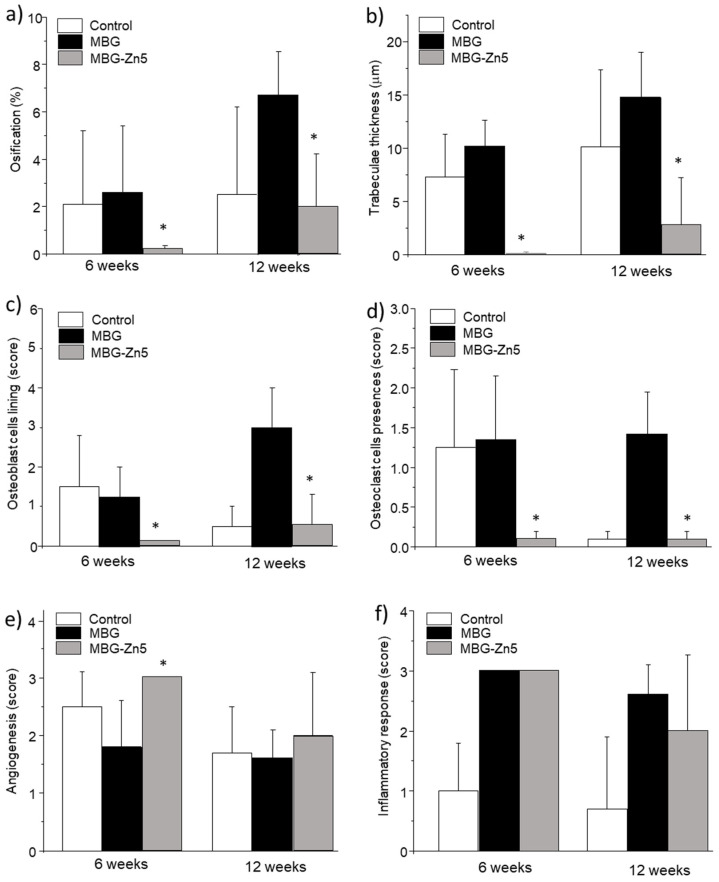
Histomorphometrical studies for the different materials implanted. (**a**) Ossification volume, (**b**) trabeculae thickness, (**c**) Osteoblast cells lining, (**d**) Osteoclast cells presence, (**e**) Angiogenesis, and (**f**) Inflammatory component. * Comparison between MBG (blank) and MBG-Zn5. Statistical significance: * *p* < 0.05.

Figure 11 shows the histomorphometric measurements obtained for control defect, MBG, and MBG-Zn5 samples 6 and 12 weeks after implantation (Figure 11a). MBG promotes a significant ossification compared to control defect 12 weeks after implantation (*p* < 0.05). On the contrary, MBG-Zn5 inhibits bone formation after 6 weeks, and no significant differences could be observed with respect to control defects. Trabeculae thickness was measured as an indicator of newly formed bone quality. No differences are observed between MBG and control defects at any time. However, the thickness of trabeculae formed in the presence of MBG-Zn5 after 12 weeks was very low and significantly thinner than that observed for MBG (*p* < 0.01). 

The presence of osteoblast cell lining after 6 weeks (Figure 11c) was mild for control defect and MBG samples, whereas no osteoblast lining could be observed in MBG-Zn5 after this period. However, MBG samples evidenced a marked presence of osteoblast cells after 12 weeks that was significantly higher than that observed for control defect and MBG-Zn5 (*p* < 0.01). The presence of osteoclasts (Figure 11d) follows a behavior similar to that of osteoblasts. Six weeks after implantation we could observe a minute presence of osteoclasts in control and MBG samples, whereas resorbing cells were absent in the case of MBG-Zn5. However, osteoclasts could be only observed in MBG samples after 12 weeks, indicating that bone remodeling is still active in the case of MBG, whereas this process stops in the case of control defects and never takes place when MBG-Zn5 is implanted. Angiogenesis was evaluated by counting the number of blood vessels by unit area of visual field. After 6 weeks, control defects and MBG-implanted bones show a moderate presence of blood vessels, whereas defect implanted with MBG-Zn5 showed significantly higher number of vessels (*p* < 0.05), although after 12 weeks the three samples showed a very similar and moderate angiogenesis. The evaluation of the inflammatory response evidenced a marked infiltration of the inflammatory component in the case of MBG and MBG-Zn5, which was significantly higher than the mild inflammatory response observed in the control defect. 

## 3. Discussion

The results obtained by XRD, TEM, and nitrogen adsorption analysis evidence that substitution of Ca^2+^ by Zn^2+^ in SiO_2_–CaO–P_2_O_5_ mesoporous glasses results in the shrinkage of the mesoporous structure, involving unit cell decrease (pore-to-pore distance), as well as a decrease of wall thickness. In addition, substitution of Ca^2+^ for Zn^2+^ cations also has a deleterious effect on the mesoporous ordering, resulting in a transition from a well-ordered hexagonal mesoporous structure for MBG to a disordered worm-like porous structure for MBG-Zn5. The structural contraction of the mesoporous bioactive glass strongly correlates with the ZnO content and can be explained in terms of the smaller ionic radii of Zn (II), 0.60 A, compared to Ca (II), 1.00 A. Moreover, the strong ionic character of Ca (II) ions is to impose their role as network modifiers of the silica network in the SiO_2_–CaO–P_2_O_5_ ternary system, disrupting the covalent network and increasing the volume. On the contrary, the partially covalent character of Zn (II) ions allows them to behave as both network formers and network modifiers [28] in such a way that the volume of the glass matrix would be significantly reduced when Ca^2+^ is partially substituted by Zn^2+^. The loss of mesoporous order in the MBG-Zn5 material indicates that Zn^2+^ cations strongly interfere in the cooperative self-assembly mechanism between the soluble SiO_2_ precursor species (network-forming species) and the F127 micelles, which is necessary for the formation of an ordered mesophase during the EISA process. This could be due to the high polarization ability of Zn^2+^ cations, which would interfere in the cooperative self-assembly process between the precursor species of the inorganic component and the protonated F127 during mesophase formation in acid media. Consequently, for the MBG-Zn5 composition, the 2D hexagonal order is lost, and a secondary and disordered porosity appears due to the incorporation of Zn^2+^ cations into the SiO_2_ network. In addition to introducing structural disorder, the presence of Zn^2+^ cations results in a significant decrease in the in vitro bioactivity of quaternary MBGs of the SiO_2_–CaO–P_2_O_5_–ZnO system. Certainly, all materials formed an amorphous calcium phosphate phase on the surface when treated with SBF. However, the crystallization of this amorphous phase into a carbonated hydroxyapatite phase is retarded by the presence of Zn^2+^ and inhibited in the case of MBG-Zn5. This deleterious effect of Zn^2+^ cations on the in vitro bioactivity of MBGs has been previously reported by different research groups [26,32,34,35,36,37], although there is some controversy on this issue [41]. Despite the initial formation of an amorphous calcium phosphate (stage 4 of the Hench bioactivity process in silica-based glasses), the presence of Zn^2+^ prevents the formation of a CHA phase. This fact can be explained in terms of the small size of Zn^2+^ cations compared to Ca^2+^, which prevents the formation of a stable hydroxyapatite phase when Zn^2+^ cations have to occupy the Ca^2+^ cation sites. Kokubo et al. stated that the bone-bonding ability of a material could be evaluated by examining the ability of apatite formation on the material when it is treated with SBF [8]. Bone bonding would result in bone healing through the osteoconduction and/or osteoinduction properties of the material. The in vitro behavior of ZnO-containing MBGs indicates that the presence of Zn^2+^ ions would hinder bone bonding under in vivo conditions, as it delays or even inhibits CHA crystallization on the surfaces of Zn-containing MBGs. However, our in vitro studies with MC3T3 osteoprogenitor cells evidence that Zn^2+^ cations are cytocompatible, stimulate differentiation of preosteoblasts, and also stimulate several genes related to osteogenesis and angiogenesis in a dose-dependent manner, which indicates that the incorporation of Zn^2+^ into MBGs would stimulate bone regeneration processes when implanted in vivo. In view of the results obtained under in vitro conditions, it seems that Zn^2+^ would play a contradictory role in bone regeneration processes mediated by MBG implantation. Whereas Zn^2+^ cations hinder the formation of the mineral phase of the bone on the MBGs surface, they simultaneously stimulate osteoprogenitor cells’ function. In order to shed light on this apparent contradiction, we decided to study the in vivo behavior of MBG and MBG-Zn5, the one with higher ZnO content, using a cavitary bone defect sheep model. In vivo studies demonstrated that the incorporation of ZnO into the MBGs inhibits the binding of bone to the material, initially generating a marked inflammatory reaction that leads to the formation of fibrous tissue. This fibrosis forms around the granules of the material, which are not degradable. This behavior differs from that of ZnO-free MBGs, which initially generate an inflammatory response that is partially replaced by new bone tissue after 12 weeks. On the other hand, the MBGs degrade without being observed in histological sections 12 weeks after implantation. These results indicate that the stability of MBG-Zn5 and the inhibition of hydroxyapatite formation prevent the bioactive behavior of these materials under in vivo conditions. The incorporation of ZnO by CaO in the SiO_2_–P_2_O_5_–CaO–ZnO system involves the substitution of Ca^2+^ ions, which are network modifiers, by Zn^2+^ ions, which can act simultaneously as network formers and network modifiers. Ion release studies indicate that initially (within 21 days), SiO_2_ dissolution is similar in both materials, although histological images after 12 weeks show that MBG-Zn5 is a much more stable material. Ion release studies also show that Zn^2+^ cations are partially solubilized, which is probably due to the release to the medium of the Zn^2+^ fraction that was incorporated as a network modifier. This release of Zn^2+^ ions to the medium would be responsible for the inhibition of hydroxyapatite formation on the glass surface. In this sense, the release of Ca^2+^ ions and the presence of phosphates allow the formation of amorphous calcium phosphate (stage 4 of the bioactive process according to Hench). However, the presence of Zn^2+^ cations, which are much smaller and polarizing than Ca^2+^ cations, prevents the crystallization of a carbonate hydroxyapatite phase (stage 5 of the Hench process), as indeed occurs in the MBG material. Despite the positive effect of Zn^2+^ on osteoprogenitor cells, our results show that without the formation of the hydroxyapatite phase on the surface of MBGs, the material will not be bioactive under in vivo conditions and rather will be encapsulated by fibrous tissue and inflammatory components from the early stages after implantation. The angiogenic character presented by Zn^2+^ ions in vitro through VEGF expression is also observed under in vivo conditions with an increased presence of blood vessels, but this angiogenesis seems to contribute only to the inflammatory response without favoring the formation of new bone tissue. Thus, the differences observed in vivo and in vitro could be due to the encapsulation of the material in vivo by fibrous tissue, which has prevented the correct diffusion of the Zn ions and their effect on bone cells. The final consequence is that the possible beneficial effects of Zn^2+^ on bone regeneration are not observed. The infiltration of inflammatory tissue and, subsequently, of fibrous tissue inhibits bone remodeling, as can be deduced from the absence of osteoblasts and osteoclasts during the entire implantation period.

## 4. Materials and Methods

### 4.1. Synthesis of Materials

MBGs of composition 70SiO_2_-5P_2_O_5_-(25-x)CaO-xZnO for x = 0, 1.25, 2.5, and 5 (% mol) were prepared by the evaporation-induced self-assembly (EISA) method. For this purpose, Pluronic^®^ F127 copolymer was employed as a structure-directing agent (SDA) and 1 M HNO_3_ as catalyst. Triethyl phosphate (TEP), tetraethyl orthosilicate (TEOS), calcium nitrate tetrahydrate, and zinc nitrate hexahydrate were used as phosphate, silica, calcium oxide, and zinc oxide precursors, respectively. All reagents were purchased from Sigma Aldrich except zinc nitrate hexahydrate, which was purchased from Panreac. First, 13.87 g of Pluronic^®^ F127 was dissolved in 180 mL of ethanol (99.98%) and 7.5 mL of HNO_3_ 1 M and kept 1 h under stirring. The amount of each precursor (Table 3) was added at 1 h intervals except for the zinc precursor, which was added after 12 h of stirring at 380 rpm and 40 °C. The resulting solution was kept 1 h more, then cast in Petri dishes and aged for 5 days at 30 °C in order to evaporate the solvent. The resulting dry laminar gel was heated for 6 h at 700 °C (heating ramp of 5 °C/min) then milled and sieved, thus obtaining particles with a size between 700 and 250 μm.

### 4.2. Characterization of MBG-Zn

In order to verify the total removal of F127 (see Figure S1 in supporting information), a thermogravimetric analyses (TG) were performed in the 30 °C to 900 °C interval (air flow: 100 mL/min) in a Perkin Elmer Pyris Diamond system (Waltham, MA, USA). The mesoporous structure of the samples was characterized by small-angle X-ray (SAXRD) diffraction in a X’pert-MPD system (Eindhoven, The Netherlands) equipped with Cu Kα radiation in the 0.6° to 8° 2θ range and transmission electron microscopy (TEM) in a JEM-1400 JEOL, which operates between 40 and 120 kV (Tokyo, Japan). Energy-dispersive X-ray analysis (EDX) was performed with a CCD camera (KeenView Camera) and operated at 120 kV in combination with TEM to determinate the sample compositions. The textural properties were characterized by nitrogen adsorption using a Micromeritics 3 Flex 8 (Norcross, GA, USA). Samples were previously degassed for 24 h at 125 °C under vacuum. Surface area, SBET, was calculated by the Brunauer–Emmett–Tellet (BET) method; meanwhile, pore size distribution was obtained from the adsorption branch of the isotherm by means of the Barrett—Joyner–Halenda (BJH) method. Bioactivity was evaluated by Fourier transform infrared spectroscopy (FTIR) in a Thermo Scientific Nicolet iS10 apparatus (Waltham, MA, USA) equipped with a SMART Golden Gate attenuated total reflection (ATR) diffuse reflectance accessory and scanning electron microscopy (SEM) in a JSM-6400 JEOL (Tokyo, Japan) operating at 25 kV coupled with an OXFORD LINK EDX.

### 4.3. In Vitro Studies

Bioactivity tests were performed by assessing the formation of an apatite-like layer on the MBGs’ surface by soaking them into simulated body fluid (SBF). Samples were sterilized by UV radiation for 20 min in a laminar flux cabinet, and the SBF was filtered with 0.22 μm filter to avoid bacterial contaminations. Following previously reported protocols [10,42], 50 mg of MBG samples were soaked in 7 mL of SBF in a polyethylene container, stirred, and kept at 37 °C for 8, 24, 48 h and 4, 7, and 15 d. The assays were performed with two replicates per time. At the end of the assay, the samples were removed and washed with acetone and ethanol to stop the apatite formation reaction. Finally, the samples were dried in an oven at 60 °C for subsequent FTIR spectroscopy and SEM studies. Ion release assay was performed by placing 25 mg of MBG samples into 5 mL of α-MEM with 1% of streptomycin–penicillin (Sigma-Aldrich, St. Louis, MO, USA) under stirring and kept at 37 °C during 24, 72 h and 7, 14, and 21 d. For this assessment, two replicas were used of each composition. Each time an aliquot was extracted, the remaining volume was removed and refilled. The cumulative concentration of calcium, silicon, and zinc was obtained, taking 3 measurements by inductively coupled plasma/optical spectrometry (ICP/OES) using an OPTIMA 3300 DV device (Perkin Elmer, Waltham, MA, USA) to do statistical analysis. 

### 4.4. In Vitro Cellular Response

In order to evaluate the in vitro cellular response to these materials, viability and differentiation studies were performed, as well as real-time PCR using preosteoblastic MC3T3-E1 mouse cells (subclone 4 CRL-2593; ETCC, Mannassas, VA, USA) as a mammal cell model. Preosteoblastic cells were cultured in supplemented medium α-MEM with 10% fetal bovine serum (FBS, Gibco, Thermo Fisher Scientific, Wilmington, DE, USA), 1% penicillin-streptomycin, and 5 mM of L-glutamine (Gibco, Thermo Fisher Scientific, Wilmington, DE, USA), washed with phosphate-buffered saline (PBS) with a pH of 7.4 (Gibco, Thermo Fisher Scientific, Wilmington, DE, USA), and collected using 5 mL of 0.25% trypsin-EDTA (Gibco, Thermo Fisher Scientific, Wilmington, DE, USA). Cell suspensions were centrifuged for 10 min at 1200 rpm. The resulting pellet was resuspended with fresh medium, and the amount of cells seeded depended on the assay performed. Regardless of the assay, cells were kept at 37 °C in a 5% CO_2_ atmosphere. 

#### 4.4.1. Viability Assay and Cell Morphology Study

Viability was determined by the Alamar Blue Method (AbD Serotec, Oxford, UK). For this assay, 2 × 10^4^ cells were seeded onto a 12-well plate. Three controls and three replicates per sample were performed and incubated for 24 h at 37 °C in a 5% CO_2_ atmosphere. After incubation, the medium was removed and refilled with a 5 mg/mL concentration of MBG in fresh medium and incubated for 1, 3, and 7 days. Thereafter, cells were washed with PBS and exposed to 1:10 solution of Alamar Blue reagent (Invitrogen, Thermo Fisher Scientific, Wilmington, DE, USA) and fresh medium for 4 h in darkness, according to the manufacturer’s instructions. Fluorescence was measured with a Synergy 4 Multimode Plate Reader (BioTek Instruments, Winooski, VT, USA) with an excitation of 560 nm and emission of 590 wavelengths at 1, 3, and 7 days. Morphology studies were carried out after the viability assays. Cells were fixed with ethanol (75% purity) for 2 min at 37 °C. Phalloidin-ATTO 565 (1:40 dilution, Molecular Probes) was added for 10 min in the dark. Then, the medium was removed and filled with Fluoroshield staining with 2-(4-amidinophenyl)-6-indolecarbamidine dihydrochloride (DAPI) (1:1000, Sigma Aldrich, St. Louis, MO, USA) for 10 min in the dark. Finally, each well was washed twice and kept in PBS until microscopic analysis. The images were obtained with an EVOS FL Cell Imaging System inverted optical microscope equipped with three types of led light (IEX (nm); IEM (nm)): DAPI (357/44; 447/60), GFP (470/22; 525/50), RFP (531/40; 593/40) from AMG (Advance Microscopy Group, Bothell, WA, USA). The red channel was used to observe the cytoskeleton and the blue to observe the cell nucleus.

#### 4.4.2. Alkaline Phosphatase (ALP) Activity

The alkaline phosphatase activity is widely known as an early marker of osteoblast differentiation and osteogenesis activity. In order to determine the effect of MBG-Zn on the ALP activity of MC3T3-E1 preosteoblasts, 7 × 10^4^ cells were seeded into a 24-well plate, with two controls and two replicates per sample. Twenty-four hours after the seeding, the medium was substituted with 5 mg/mL of MBG in differentiation medium, 0.05 mg/mL of l-ascorbic acid 2-phosphate (Sigma-Aldrich, St. Louis, MO, USA), 2.18 mg/mL β-glycerophosphate (Sigma-Aldrich, St. Louis, MO, USA), 1% penicillin/streptomycin (Gibco, Thermo Fisher Scientific, Wilmington, DE, USA), 5 mM of L-glutamine (Sigma-Aldrich, St. Louis, MO, USA), and 10% FBS (Gibco, Thermo Fisher Scientific, Wilmington, DE, USA). Cells were maintained for 7 days at 37 °C under 5% CO_2_, with the differentiation culture medium being renewed after 3 days. ALP activity was measured using the Reddi and Huggins method based on the hydrolysis of p-nitrophenylphosphate to p-nitrophenol. This measurement was normalized to the cell protein content, inferred by the Bradford’s method using bovine serum albumin as standard. 

#### 4.4.3. Real-Time PCR

In order to research the genetic expression of osteoblastic differentiation, 2 × 10^3^ cells were seeded into a 6-well plate, with two controls and two replicates per sample. Then, 5 mg/mL of MBG in differentiation medium was added 24 h after the seeding as previously reported. Cells were cultured for 10 days at 37 °C under 5% CO_2_, refilling differentiation culture medium every 3 days. The gene expression of the osteoblastic differentiation marker and angiogenic factor (RUNX2, OPG, and VEGF, respectively) were quantified by real-time PCR using QuantStudio5 equipment and a previously described protocol (Applied Biosystem-Thermo Scientific, Foster City, CA, USA). TaqManMGB probes were obtained by Assay-by-DesignSM (applied Biosystems). For each sample and using the given cycle threshold (Ct) value, mRNA copy numbers were calculated and glyceraldehyde-3-phosphate dehydrogenase (GAPDH) rRNA (a housekeeping gene) was amplified in parallel with the tested genes. The number of amplification steps required to reach an arbitrary Ct was computed. The relative gene expression was represented by 2^−ΔΔCt^, where ΔΔCt = ΔCt_target_ gene − ΔCt_GAPDH_. The fold change for the treatment was defined as the relative expression compared with the control GAPDH expression, calculated as 2^−ΔΔCt^, where ΔΔCt = ΔC_treatment_ − ΔC_control_. 

### 4.5. In Vivo Studies

Twelve adult female Merino sheep were included in the study. The animals were randomly divided into two groups, based on different endpoints to better study biomaterial osseointegration: 6 weeks (*n* = 6) and 12 weeks (*n* = 6). Animals from the 6-week study had a mean preoperative weight of 43.67 ± 6.11 kg and animals from the 12-week study weighed 49.92 ± 8.43 kg. 650 mg of the biomaterials (MBG (control material) and MBG-Zn5) were blindly implanted in all sheep under aseptic conditions and general anaesthesia (see Figure S2 in supporting information). Sheep were induced with propofol (4 mg/kg) and maintained on isoflurane (1.5%) in oxygen. Animals were intraoperatively administered meloxicam (0.4 mg/kg), buprenorphine (0.01 mg/kg), and ceftiofur (1 mg/kg). Five cylindrical critical-size defects (8 × 12 mm) were created in each sheep by drilling the cancellous bone under continuous irrigation with cold sterile saline of the greater tuberosity of both humeri, the proximal tibia epiphysis bilaterally, and the medial epicondyle of the left femur. In the 6-week and 12-week study animals, 6 samples of each biomaterial were implanted, and 6 defects were left empty as control in each group. Once the biomaterials were implanted, the muscular and subcutaneous tissue was approximated with absorbable monofilament sutures and the skin with absorbable braided sutures. Postoperative analgesia was maintained with buprenorphine (0.01 mg/kg/12 h/3 days) and meloxicam (0.4 mg/kg/24 h/7 days). Ceftiofur (1 mg/kg/24 h) was administered for 7 days as prophylactic antibiotherapy. The health condition of all animals was checked daily along the whole study by an accredited veterinarian. Immediately after the surgical procedure and before the sample removal, a computed tomography (CT) scan was performed (see Figure S3 in supporting information). The bone samples containing the implants or defect areas were harvested and fixed in 96% ethanol 6 and 12 weeks after the implantation, depending on the endpoint of each group. 

### 4.6. Histological Studies

Six and twelve weeks after implantation, the bone segments containing the defect were removed and fixed by immersion in 96% ethanol. Bone segments were processed as described elsewhere [13], and histological sections were stained with Hematoxylin-Eosin (Agilent Dako Coverstainer for H&E). Images were obtained with a Leica DMD1008 microscope and an Olympus BX40 microscope and analyzed with ImageJ 1.x to calculate the bone ingrowth area, trabeculae thickness, and number of blood vessels. For the evaluation of the angiogenesis, inflammatory component, and osteoclast presence, we used a four-grade scale based on the density and distribution (absent/mild/moderate/marked, scored 0 to 3). Multinucleated cells situated in a resorption lacuna were identified as osteoclasts, and oval cells with abundant blue-grey cytoplasm and perinuclear hofs surrounding the forming bone were identified as osteoblasts (see Table S1 in supporting information). 

### 4.7. Statistical Analysis

Results were expressed as mean ± standard error of mean (SEM). Statistical evaluation was carried out with a Student’s *t*-test. All statistical tests were conducted at the two-sided 0.05 (*p*-value) level of significance.

## 5. Conclusions

Mesoporous bioactive glasses in a system of (% mol) 70 SiO_2_-5 P_2_O_5_-(25-x) CaO-x ZnO were synthesized by the EISA method and evaluated under in vitro and in vivo conditions. The incorporation of ZnO leads to physical–chemical changes that comprise shrinkage of the ordered mesoporous structure and decrease of surface area and porosity. In addition, Zn content hampers the in vitro bioactivity by inhibiting the formation of a carbonate hydroxyapatite phase when MBGs are in contact with simulated body fluid. The incorporation of ZnO in MBGs stimulate the in vitro proliferation and differentiation of MC3T3-E1 osteoprogenitor cells in a concentration-dependent manner. Futhermore, the expression of osteogenic and angiogenic genes is increased for those compositions with higher ZnO contents. In vivo studies in a sheep model evidence that, despite the excellent in vitro response of osteoprogenitor cells to the ZnO content, the mineralization inhibition by Zn^2+^ cations hinder the in vivo bioactivity of ZnO containing MBGs.

## Figures and Tables

**Table 1:** Chemical composition (% mol) calculated by EDX spectroscopy.

Sample	SiO_2_ (% mol)	CaO (% mol)	P_2_O_5_ (% mol)	ZnO (% mol)
MBG	69.8 (±3.6)	25.5 (±2.6)	4.7 (±1.7)	0
MBG-Zn1	74.1 (±5.6)	22.6 (±3.1)	2.9 (±1.4)	0.4 (±0.3)
MBG-Zn3	71.4 (±5.3)	19.5 (±6.9)	5.1 (±1.9)	4.0 (±0.3)
MBG-Zn5	70.7 (±5.4)	19.3 (±4.2)	4.5 (±1.6)	5.5 (±1.7)

**Table 2:** Textural properties calculated by nitrogen adsorption analysis.

Sample	Surface Area (m^2^/g)	Pore Volume (cm^3^/g)	Pore Size (nm)	a_0_ *^a^* (nm)	Wall Thickness *^b^* (nm)
MBG	300.6	0.38	5.90	10.2	5.1
MBG-Zn1	271.5	0.33	5.26	9.9	3.6
MBG-Zn3	204.9	0.25	4.87	8.0	3.1
MBG-Zn5	207.1	0.20	4.33	NA	NA

*^a^* Calculated by XRD as a_0_ = d_(1 0 0)_·2/√3; *^b^* Calculated as a_0_—pore size.

**Table 3:** Amounts of reagents used during synthesis of the Zn-MBGs.

Sample	MBG Nominal Composition (% mol)	TEP (mL)	TEOS (mL)	Ca(NO_3_)_2_ (g)	Zn(NO_3_)_2_ (g)
MBG	70 SiO_2_-5 P_2_O_5_-25 CaO	2.904	26.7	10.002	----
MBG-Zn1	70 SiO_2_-5 P_2_O_5_-23.7 CaO-1.3 ZnO	2.904	26.7	9.588	0.635
MBG-Zn3	70 SiO_2_-5 P_2_O_5_-22.5 CaO-2.5 ZnO	2.904	26.7	9.084	1.26
MBG-Zn5	70 SiO_2_-5 P_2_O_5_-20 CaO-5 ZnO	2.904	26.7	8.001	2.541

## Data Availability

Not applicable.

## Supplementary Materials

The following supporting information can be downloaded at: https://www.mdpi.com/article/10.3390/ijms232213918/s1.
