# Supplementary material for: In Vitro and In Vivo Response of Zinc-Containing Mesoporous Bioactive Glasses in a Sheep Animal Model
Source: Int J Mol Sci. 2022 Nov 11;23(22):13918. doi: 10.3390/ijms232213918 (PMC9698899; doi:10.3390/ijms232213918)
Supplement: Supplementary file 1 [file ijms-23-13918-s001.zip › ijms-2007611-SI.pdf]

# In vitro and in vivo response of zinc-containing mesoporous bioactive glasses in a sheep animal model

# Supporting information

S.1. Thermogravimetrical analysis.

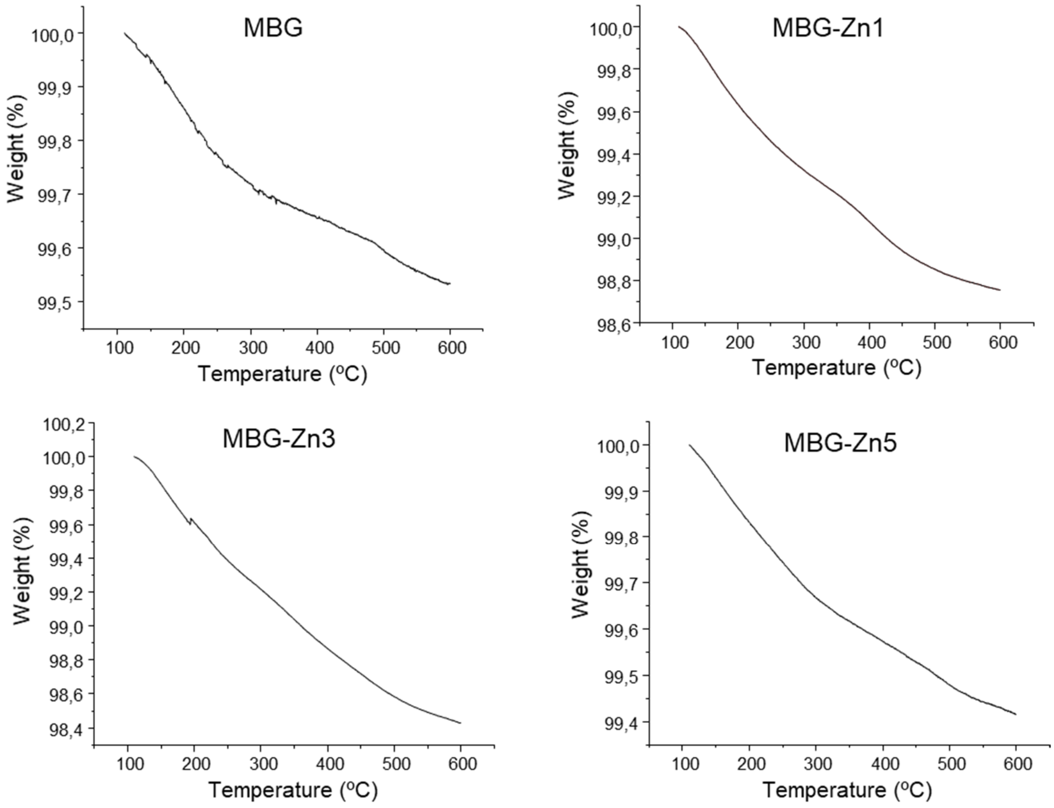


**Figure S1.** Thermogravimetrical analysis of the different materials synthesized. The weight losses observed range between 0.4 and 1.5 % and would correspond to the condensation of silanol groups.

S.2. Implantation procedure

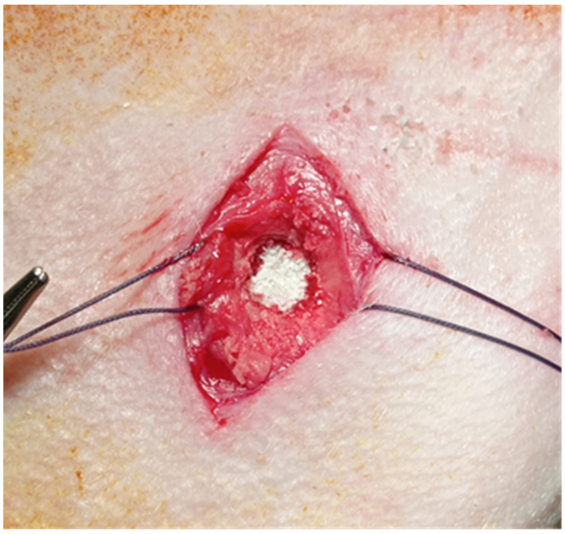

**Figure S2.** Image of the bone defect filled with MBG-Zn5 granules.

S.3. Computed tomography (CT) images

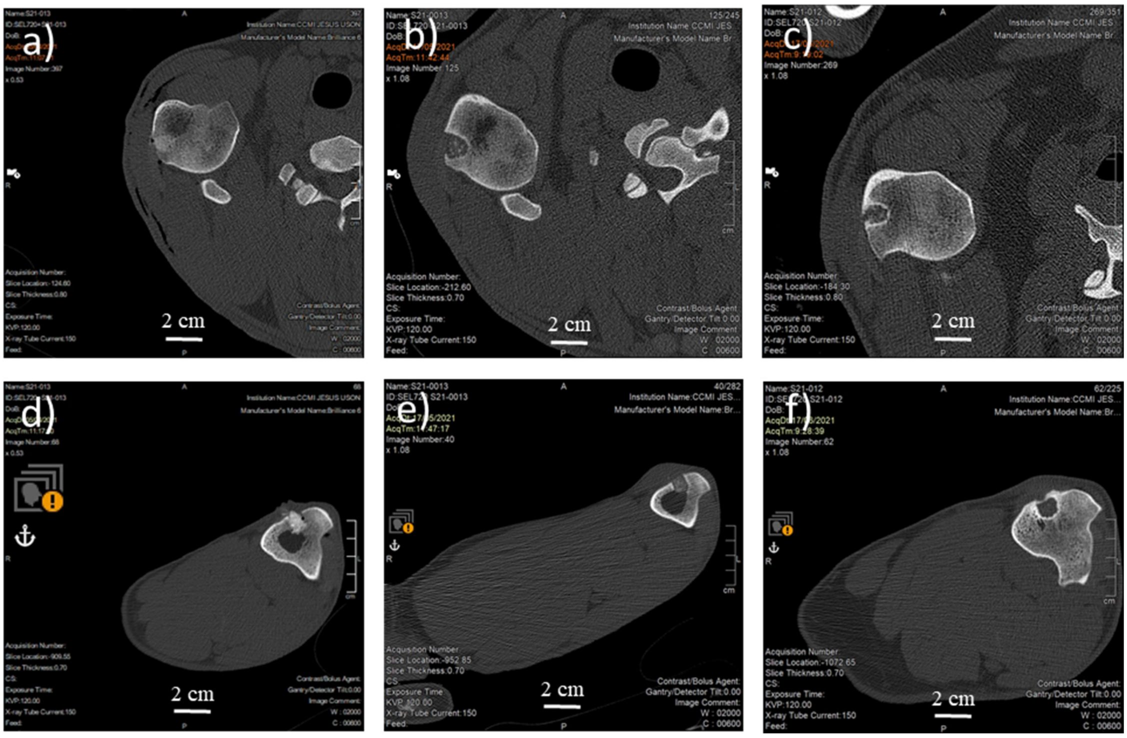


**Figure S3.** CT images for a) MBG post implantation, b) MBG after 6 weeks, c) MBG after 12 weeks, d) MBG-Zn5 post implantation, e) MBG-Zn5 after 6 weeks and f) MBG-Zn5 after 6 weeks.

S.4. Cells morphology studies

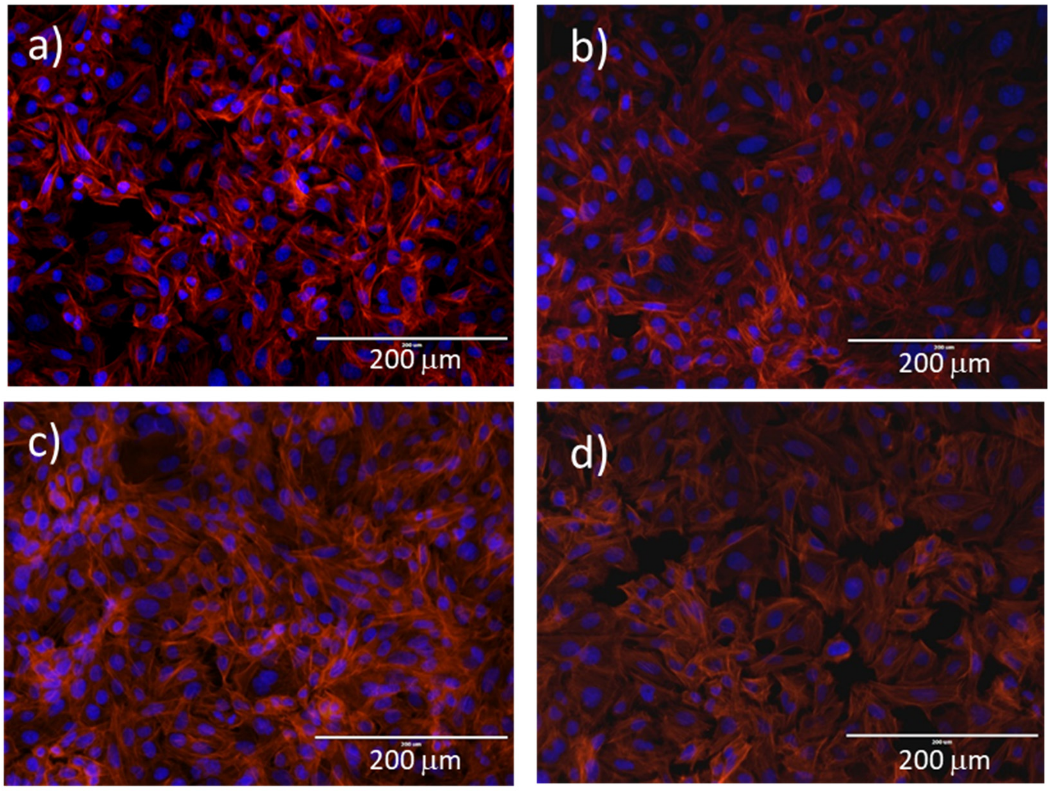


**Figure S4.** Optical images of MB3T3-E1 pre-osteoblast cells obtained after 1 week of culture in the presence of a) MBG; b) MBG-Zn1; c) MBG-Zn3 and d) MBG-Zn5

S.5. Apatite-forming ability of MBG in α-MEM culture media

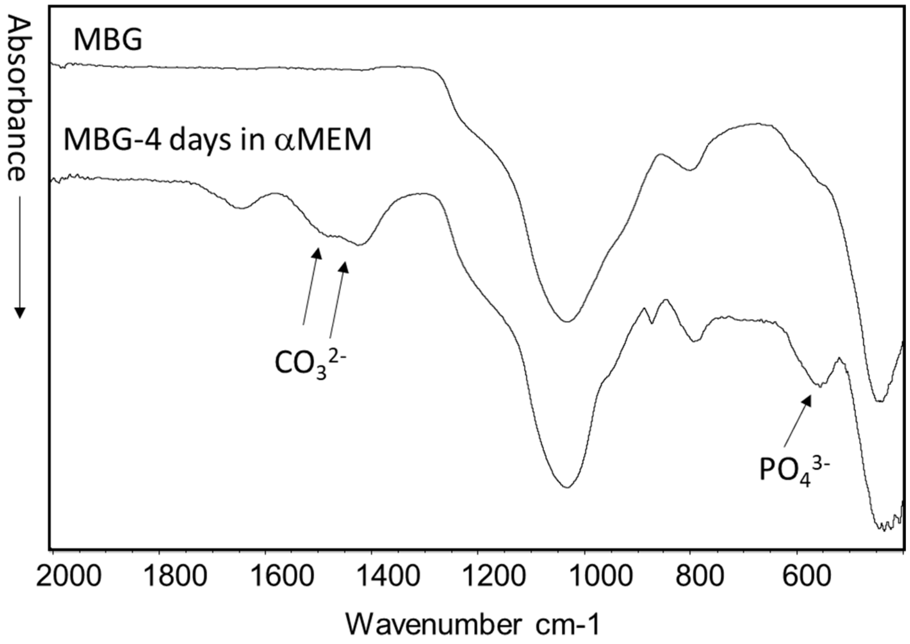


**Figure S5.** FT-IR spectrum of MBG sample before and after being soaked in α-MEM for 4 days. The presence of absorption bands at 560, 1419 and 1455 $cm^{-1}$ evidence the formation of a new carbonated calcium phosphate phase similarly to the one observed when soaked in SBF

**Table S1.** Guide for quantitative histological analysis

| Scoring for the extent of inflammatory component | |
|---|---|
| Description | Score |
| Absent | 0 |
| Mild. Mild or moderate focal infiltrate, or mild multifocal infiltrate | 1 |
| Moderate. Marked focal, moderate or marked multifocal, or mild diffuse infiltrate grade | 2 |
| Marked. Marked diffuse infiltrate | 3 |

| Scoring for the extent of blood vessels formation | |
|---|---|
| Description | Score |
| Absent | 0 |
| Mild: 0-5 vessels /20X microscope field | 1 |
| Moderate: 5-10 vessels /20X microscope field | 2 |
| Marked >10 vessels / 20X microscope field | 3 |

| Scoring for the extent of osteoblasts presence | |
|---|---|
| Description | Score |
| 0 % of the new formed trabeculae contain osteoblasts | 0 |
| 1-25 % of the new formed trabeculae contain osteoblasts | 1 |
| 26-50 % of the new formed trabeculae contain osteoblasts | 2 |
| 51-75% of the pores contain osteoblasts | 3 |
| 76-100 % of the pores contain osteoblasts | 4 |

| Scoring for the extent of osteoclasts presence | |
|---|---|
| Description | Score |
| Absent | 0 |
| Mild: 1 – 5 osteclasts /20X microscope field | 1 |
| Moderate: 5 - 10 osteclasts /20X microscope field | 2 |
| Marked: More than 10 osteoclasts/ 20X microscope field | 3 |
